# Optimal adjustment sets for causal query estimation in partially observed biomolecular networks

**DOI:** 10.1093/bioinformatics/btad270

**Published:** 2023-06-30

**Authors:** Sara Mohammad-Taheri, Vartika Tewari, Rohan Kapre, Ehsan Rahiminasab, Karen Sachs, Charles Tapley Hoyt, Jeremy Zucker, Olga Vitek

**Affiliations:** Khoury College of Computer Sciences, Northeastern University, Boston, MA 02115, USA; Khoury College of Computer Sciences, Northeastern University, Boston, MA 02115, USA; Khoury College of Computer Sciences, Northeastern University, Boston, MA 02115, USA; Google, Cambridge, MA 02142, USA; Next Generation Analytics, Palo Alto California, USA; Modulo Bio Inc, Los Altos, California, USA; Answer ALS, New Orleans, LA, USA; Laboratory of Systems Pharmacology, Harvard Medical School, Boston, Massachusetts, USA; Pacific Northwest National Laboratory, Richland, Washington 99354, USA; Khoury College of Computer Sciences, Northeastern University, Boston, MA 02115, USA

## Abstract

Causal query estimation in biomolecular networks commonly selects a ‘valid adjustment set’, i.e. a subset of network variables that eliminates the bias of the estimator. A same query may have multiple valid adjustment sets, each with a different variance. When networks are partially observed, current methods use graph-based criteria to find an adjustment set that minimizes asymptotic variance. Unfortunately, many models that share the same graph topology, and therefore same functional dependencies, may differ in the processes that generate the observational data. In these cases, the topology-based criteria fail to distinguish the variances of the adjustment sets. This deficiency can lead to sub-optimal adjustment sets, and to miss-characterization of the effect of the intervention. We propose an approach for deriving ‘optimal adjustment sets’ that takes into account the nature of the data, bias and finite-sample variance of the estimator, and cost. It empirically learns the data generating processes from historical experimental data, and characterizes the properties of the estimators by simulation. We demonstrate the utility of the proposed approach in four biomolecular Case studies with different topologies and different data generation processes. The implementation and reproducible Case studies are at https://github.com/srtaheri/OptimalAdjustmentSet.

## 1 Introduction

Causal query estimation (Pearl 2009) is an essential task in analysis of biomolecular networks. Queries of the form ‘When we intervene on X, what is the effect on its descendent Y?’ provide insights into the function of biomolecular systems, enable medical decision making, and help develop drugs. Applying an intervention and collecting interventional data may be challenging or expensive. Thus methods have been proposed for estimating causal queries from observational data without interventions (Jung *et al.* 2020a,b).

Causal query estimation from observational data relies on a known biomolecular network, i.e. a graph where variables (nodes) are signaling proteins, genes, transcripts or metabolites, and directed edges are previously established causal regulatory relationships. Such networks are available in knowledge bases such as INDRA (Bachman *et al.* 2022), Reactome (Gillespie *et al.* 2022), or Omnipath (Türei *et al.* 2016). Estimation of causal queries with the entire biomolecular networks can be computationally intractable. Perhaps counterintuitively, using the full set of variables can in fact be harmful, and lead to bias, or increase variance (Cinelli *et al.* 2020). Therefore, causal query estimation should rely on a well-chosen subset of variables that minimize the bias and the variance of the estimator.

Collecting observational data on the network variables incurs highly varying costs. For example, while developing a new antibody for flow cytometry experiments is expensive, quantifying a protein with an existing antibody is cheaper. In mass spectrometry, measurements of low-abundant or modified proteins may be more costly than when proteins are highly abundant. Quantifying the entire transcriptome with RNA sequencing may be more expensive than quantifying its subset, e.g. with qPCR. Thus, the measurement cost is an important consideration, and typically networks are only partially observed (i.e. some of its nodes are latent).

A common procedure for selecting a subset of network variables is covariate adjustment (Pearl 2009). The procedure relies on selecting ‘valid adjustment sets’, i.e. the network variables that, if left unadjusted for, induce biased estimation. However, estimators employing different valid adjustment sets have different variances. Existing approaches for selecting optimal adjustment sets, i.e. a valid adjustment set with the least asymptotic variance, rely on network topology. While such criteria are clear for models with no latent variables (Henckel *et al.* 2019; Rotnitzky and Smucler 2020), they are only applicable to a narrow class of network topologies for models where latent variables exist (Runge 2021; Rotnitzky and Smucler 2022).

Even when the topology-based methods are applicable to partially observed biomolecular networks, their practical utility is quite limited, for two reasons. First, the asymptotic criteria are rarely applicable in experiments with limited number of biological replicates. Second, many biomolecular networks share topology, but differ in processes that generate the observational data. In these cases, the topology-based criteria fail to distinguish the variances of the adjustment sets. This can lead to sub-optimal adjustment sets, and to miss-characterization of the effect of the intervention.

We propose an approach for determining ‘optimal adjustment sets’ in terms of bias, small-sample variance, and measurement cost. The approach empirically learns the data generating processes from historical experimental data. It then characterizes the bias, variance, and measurement cost of the estimator by simulating observations from the learned data generation process, and by evaluating the estimator.

Simulation-based inference, such as in this manuscript, has a long and successful history in computational biology, e.g. single-cell RNA-seq data simulation (Marouf *et al.* 2020; Cao *et al.* 2021; Sun *et al.* 2021), stochastic simulation of biochemical reaction systems (Marchetti *et al.* 2017), high-throughput sequencing data simulations (ReSeq) (Schmeing and Robinson 2021), and genetic data simulators (Peng *et al.* 2015). Simulations have been used to model biological systems accurately, robustly, and reproducibly (Marouf *et al.* 2020), in particular in situations with unattainable ground truth (Cao *et al.* 2021; Sun *et al.* 2021; Parikh *et al.* 2022). They also help in multi-criteria decision making by examining trade-offs (Dunke and Nickel 2021).

The proposed approach uses simulations to effectively explore all the valid adjustment sets. In additions to the simulations, it uses sensitivity analysis to characterize the bias and variance of different simulation strategies and different causal query estimators in the presence of latent variables. The overall strategy allow us to find the optimal adjustment sets when they cannot be found based on graphical criteria, or when the number of data points is small. Moreover, it allows comparison of the valid adjustment sets.

We demonstrate the effectiveness of the proposed approach in four Case studies. In the first two, simulated models were constructed based on standard biological practice and used as ground truths to compare against the fitted models. In the remaining two Case studies, past observational experimental data were used to generate synthetic data, and experimental interventional data were used to verify the validity of the results.

## 2 Background

### 2.1 Graphical notation and causal inference

Let boldface letters such as X be a set of random variables and non-boldface letters such as *X* be a random variable. Let *x* be an instance of *X*, and x an instance of X. Let G=(V∪U,E) be a directed acyclic graph (DAG) as in Fig. 1, where V are observable variables (white nodes), and U are latent (grey nodes), and E is a set of edges.

**Figure 1.**
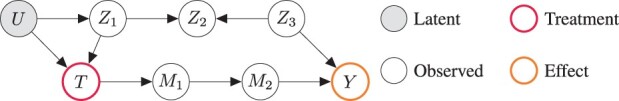
A directed acyclic graph (DAG). $Z_{2}$ is a collider on the backdoor path $T\leftarrow Z_{1}\to Z_{2}\leftarrow Z_{3}\to Y$.

An **intervention** (perturbation) on a target variable in the graph (treatment) *T* fixes it to a constant value *t* (denoted do(T=t)), and makes it independent of its causes (Spirtes *et al.* 2000; Eberhardt and Scheines 2007).

A **causal query**$Q_{G}$ over the effect *Y* is any probabilistic query that conditions on an intervention, such as $Q_{G}=E[Y|do(T=t)]$ or E[Y|do(T=1)]−E[Y|do(T=0)]. The latter is a special case of causal query for binary treatments called **average treatment effect (ATE)** (Imbens and Rubin 2015). A causal query $Q_{G}$ is **identifiable** if the query can be estimated from the available data and the assumptions regarding the causal structure of the system (Pearl 2009). An **unidentifiable** causal query cannot be estimated using the data and the assumptions.

A **path** between two variables *T* and *Y* exists if there is a sequence of edges connecting *T* to *Y*. A **directed path** follows the direction of the edges. A **causal path** from *T* to *Y* is a directed path from *T* to *Y*. Let cp(T,Y,G) be all the variables on causal paths from *T* to *Y* in *G* excluding *T*. E.g. in Fig. 1, $cp(T,Y,G)=\{M_{1},M_{2},Y\}$.

**d-separation** (Pearl 2009) captures true independencies (i.e. separations) in a path. A set of variables *X* is d-separated from another set of variables *Y*, on a path *p*, by a (possibly empty) set of variables *Z* if and only if all of the paths between (any node in) *X* and (any node in) *Y* are blocked by *Z*. A path *p* is d-separated or blocked by a set of variables Z if and only if, (1) there is a collider that has not been conditioned on, and (2) there is a fork or chain that has been conditioned on. A path with arrows coming into both *T* and *Y* is called a **backdoor path**. A set Z d-separates *T* from *Y* if and only if Z blocks every path from *T* to *Y*. For example in Fig. 1, any subset of $\left\{Z_{1},Z_{3}\right\}$ blocks the backdoor path $T\leftarrow Z_{1}\to Z_{2}\leftarrow Z_{3}\to Y$.

Frequently, some variables in a DAG are unobserved (i.e. latent) such as *U* in Fig. 2a. DAGs with latent variables are compactly represented by **acyclic directed mixed graphs** (ADMGs) (Richardson *et al.* 2017) such as in Fig. 2c. An ADMG $G=(V,E_{d},E_{b})$ consists of a set of observable variables V, a set of directed edges $E_{d}$, and bidirected edges $E_{b}$. A bidirected edge indicates a path including any number of latent variables without colliders that point to two observable variables. ADMGs allow us to misspecify the number and type of the latent variables, as long as we accurately represent the topology over the observed variables. ADMG is the main graph structure in this manuscript.

**Figure 2.**
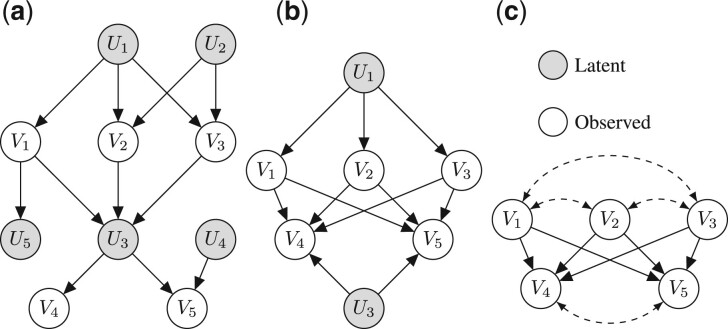
(a) DAG *G*. (b) Simplified DAG $\tilde{G}$ according to simplification rules. (c) Corresponding ADMG G. Bi-directed edges show presence of latent variable. The exact number and structure of latent variables can be misspecified in an ADMG.

An ADMG is constructed by using the following **simplification rules** (Evans 2016):

1. Remove latent variables with no children from the graph. E.g. $U_{5}$ in Fig. 2a is removed in Fig. 2b.
2. Transform a latent variable with parents to an exogenous variable where all its parents are connected to its children. E.g. $U_{3}$ in Fig. 2a and b.
3. Remove an exogenous latent variable that has at most one child. E.g. $U_{4}$ in Fig. 2a is removed in Fig. 2b.
4. If *U*, *W* are latent variables where children of *W* are a subset of children of *U*, then, *W* can be removed. E.g. $U_{1}$ and $U_{2}$ in Fig. 2a, and $U_{2}$ is removed in Fig. 2b.

### 2.2 Input variables for causal query estimators

Given an ADMG and a causal query of interest, our objective is to determine the variables in the DAG that are best used as input to a causal query estimator. The choice affects the estimator’s bias and variance. An estimator is **biased** if it systematically deviates from its target. **Variance** of the estimator is its variability across repeatedly collected data.

The set of variables blocking the backdoor paths is called a **valid adjustment set** (e.g. $\left\{Z_{1},Z_{3}\right\}$, or $\left\{Z_{1}\right\}$, or $\left\{Z_{2}\right\}$, or empty set in Fig. 1). The smallest set of variables that block all the back-door paths is called a **minimal adjustment set** (e.g. empty set in Fig. 1). However, different adjustment sets produce estimators with different variance.

A minimal subset of the variables that blocks all back-door paths and maximally reduces the variability in *Y* is an **optimal adjustment set** (e.g. $Z_{3}$ in Fig. 1). In absence of latent variables, (Rotnitzky and Smucler 2020) presented a straightforward graphical criteria for computing the optimal adjustment set. A valid, minimal, or optimal adjustment set can be computed with open-source R packages such as pcalg (Kalisch *et al.* 2012), dagitty (Textor *et al.* 2016), and causaleffect (Tikka and Karvanen 2017).

In presence of latent variables, (Runge 2021) showed that an optimal adjustment set exists in the specific case of linear models and under specific conditions. (Rotnitzky and Smucler 2022) proved the existence of an optimal adjustment set and of an efficient minimum cost adjustment set for non-linear models and specific graphical structures. Beyond that, a unique optimal adjustment set does not exist in most of the graphical structures, e.g. in structures with non-ancestors of the treatment and effect such as $Z_{2}$ in Fig. 1. This is due to the fact that, in presence of latent variables, the optimal adjustment set can depend on the data generation process. Since a same graphical structure can support multiple data generating processes, a graphical criterion alone cannot determine the optimal adjustment set. This motivated the simulation-based approach in this manuscript.

### 2.3 Simulation-based inference

Synthetic data representative of a particular experiment is an increasingly popular strategy for experiment planning, analysis, and validating causal inference methods (Parikh *et al.* 2022). Generative models such as GANs (Goodfellow *et al.* 2020) successfully generated high-quality artificial genomes (Yelmen *et al.* 2019), MRI scans (Pawlowski *et al.* 2020), and electronic health records while mitigating privacy concerns (Choi *et al.* 2017; Weldon *et al.* 2021).

Numerous single-cell RNA (scRNA-seq) sequencing data simulation methods (Marouf *et al.* 2020) have been developed to generate realistic scRNA sequencing data, and to produce data with desired structure (such as specific cell types or subpopulations) while capturing gene correlations, and generating any number of cells with varying sequencing depths (Cao *et al.* 2021; Sun *et al.* 2021). They are also useful to augment sparse cell populations and improve the quality and robustness of downstream classification. To the best of our knowledge, there are currently no approaches for simulation-based selection of optimal adjustment sets.

### 2.4 Existing causal query estimators

Given an ADMG, a causal query of interest, and an observational data, the next objective is to choose an estimator for the query. Many estimators are implemented in open-source libraries such as DoWhy (Sharma *et al.* 2019), Ananke (Bhattacharya *et al.* 2020), AIPW (Zhong *et al.* 2021), and the $Y_{0}$ engine (Zucker and Hoyt 2021).

**Non-parametric and semi-parametric estimators** such as inverse probability weight (IPW), and augmented IPW (AIPW) are asymptotically unbiased, do not require parametric assumptions, and are computationally cheap. However, they are limited to causal queries with one treatment and one effect, and the treatment must be binary-valued.

**Linear regression estimator** regresses *T* and the adjustment set on *Y*. For example in Fig. 1,


(1) $$Y=\beta_{0}+\beta T+\gamma Z_{i}+\epsilon ;\epsilon \overset{\mathrm{iid}}{∼}N(0,\sigma^{2}).$$

The coefficient β of *T* is the ATE. The coefficient γ is the effect of the adjustment variable $Z_{i}$ on the outcome *Y*. The least squares estimator $\hat{\beta }$ is an unbiased estimator of the ATE, as $Z_{i}$ blocks the backdoor path. Including more than one $Z_{i}$, i∈{1,3} from Fig. 1 does not introduce the bias in $\hat{\beta }$ (because the backdoor path remains blocked), but affects its variance. E[Y|do(T=t)] is estimated by substituting $\hat{\beta }$ and $\hat{\gamma }$ in (1) and averaging over the values of *Y*.

The linear regression approach is simple, fast, and highly interpretable. It takes as input treatment, effect, and a valid adjustment set. However, it is only appropriate when the true relationship between variables is approximately linear.

## 3 Methods

Figure 3 and Algorithm 1 overview the proposed approach.

Algorithm 1 generateSetOfRankedAdjSets

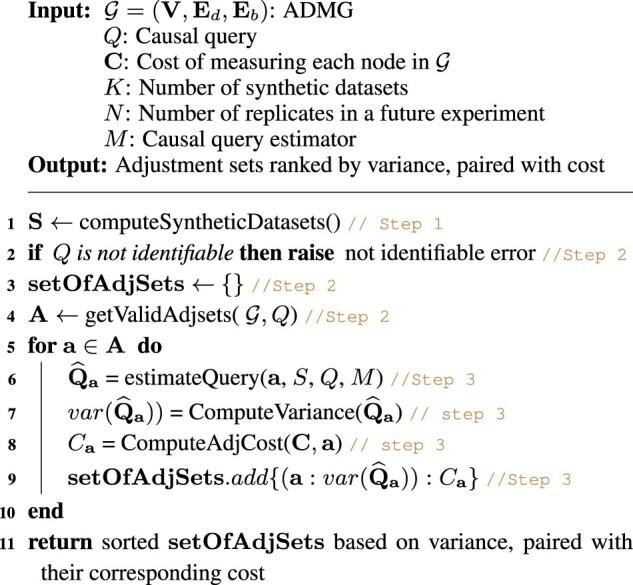

**Figure 3.**
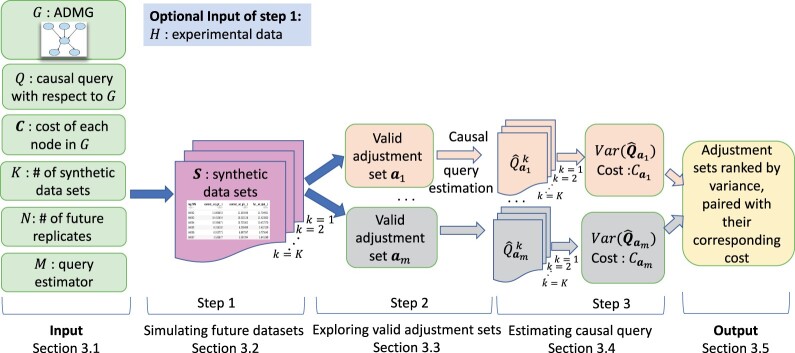
Overview of the proposed approach. Arrows indicate sequence of steps. Inputs specific to the biological problem are shown in green. Additional tuning parameters and analytical options specified for each step are shown in blue.

### 3.1 Input

The proposed approach takes as input an ADMG *G*. The ADMG is built by applying the simplification rules in Sec. 2.1 to any acyclic network, extracted from a knowledge base such as INDRA or Omnipath. There are no restrictions on the organism, or on the type of the biomolecular network. The ADMG can misspecify the structure of the latent variables, as long as it accurately represents the structure over the observable variables.

The proposed approach takes as input causal query *Q* with respect to *G* of the form E[Y|do(T=t)] or ATE=E[Y|do(T=t+1)]−E[Y|do(T=t)] for a given *t*. It also takes as input a numeric vector *C* quantifying the relative cost of experimental measurements of each node in *G*. The proposed approach has two tuning parameters, the number *K* of synthetic datasets to be simulated, and the number *N* of replicates in a future experiment. Finally, it takes as input a causal query estimator *M*, such as the estimators in Sec. 2.4.

### 3.2 Step 1: simulating future datasets

We evaluate the estimators based on different adjustment sets, by generating *K* synthetic datasets *S* that mimic the future experiment, with *N* observations each. The methodological challenges are the choice of the simulator, and the diagnostics of the simulation accuracy. When historical experimental data (e.g. prior data from the same organism, or from related systems or technologies) exist, the synthetic data is constructed based on, e.g. Tabular GAN (Xu *et al.* 2019), or statistical associations such as Bayesian network forward simulation (Koller and Friedman 2009). The latter requires as input the knowledge of the ADMG. The quality of the synthetic data is evaluated by comparing their marginal and the joint distributions to those of the experimental data. In absence of historical data, the synthetic datasets are constructed using reasonable or known ranges for model parameters, and functional forms such as Hill equations (Alon 2019). The quality is evaluated by sensitivity analysis across multiple values of parameters and functional forms.

### 3.3 Step 2: exploring adjustment sets

The inputs to the step are the ADMG *G*, and the causal query of interest *Q*. Since unidentifiable queries do not have unbiased estimators, the algorithm raises an error for unidentifiable queries (line 2). Next (line 4), the step relies on the existing algorithms for determining all the valid adjustment sets of the ADMG, such as the ones implemented in dagitty (Sec. 2.2).

In Algorithm 1, line 4 is of exponential complexity $O(2^{n})$, where *n* is the total number of valid adjustment sets. Despite the complexity, it supports many practically relevant queries. The algorithms determining all the valid adjustment sets remove from consideration all the descendants of the outcome (because the outcome is not affected by its descendants). As the result, the size of the ADMG considered by these algorithms may be substantially smaller than the input ADMG.

### 3.4 Step 3: estimating causal query

The inputs to Step 3 are the synthetic datasets *S* (output of Step 1), the valid adjustment sets A (output of Step 2), the causal query *Q*, the cost *C*, and the query estimator *M*.

Algorithm 1 iterates over all the valid adjustment sets (line 5). For each valid adjustment set, this step reports the query estimate over the *K* synthetic datasets (line 6), computes their empirical variance (line 7), and the cost of measuring the adjustment set (line 9). Finally, it adds the triple to a list for storing the results (line 9).


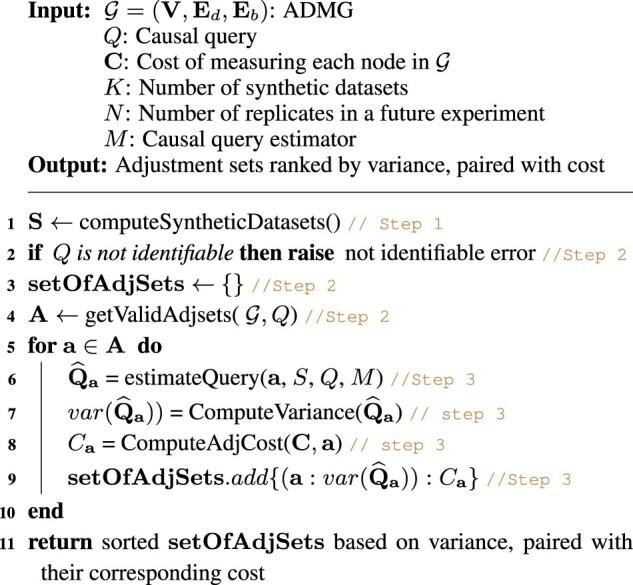


### 3.5 Output

The algorithm outputs valid adjustment sets $a_{1},\ldots ,a_{m}$ ranked according to the empirically evaluated variance of the causal query estimators. Each valid adjustment set is paired with its corresponding measurement cost. As the result, the user can design a future experiment that trades off the variance of a valid adjustment set and its experimental cost. We can evaluate the impact of the sample size and of the estimator by applying Algorithm 1 to different causal query estimators and different numbers of biological replicates *N*.

## 4 Case studies of biomolecular networks

We illustrate the practical utility of the proposed algorithm in four Case studies with a broad set of network topologies, causal query types, data generation processes and causal query estimators. Case studies 3 and 4 are based on observational experimental measurements of *Escherichia coli*, validated using interventional experimental measurements. In all the Case studies, the number of biological replicates *N* in the synthetic datasets was set to be equal to the number of biological replicates in the historical dataset. The quality of the synthetic data was evaluated by comparing their marginal and joint distributions with those of the historical experimental data (see supporting GitHub repository for detail). The Case studies took between 1 min and 1 h on a Google cloud Platform with 1 vCPU and 8 GB memory.

### 4.1 Case study 1: the illustrative example

**Overall objective:** This simple example illustrates the importance of using the proposed approach in a case where the optimal adjustment set depends on the data generation process, and cannot be found based on a graphical criterion. Further, it shows its robustness across different estimators.

**Ground truth:** Consider the weighted ADMG in Fig. 4a, where *T* is a binary treatment and *Y* is a continuous effect. In biomolecular pathways, such structures abound. Genes and proteins may be downstream of multiple regulators, such as transcription factors and signaling molecules. Colliders (non-ancestors of the treatment and effect) such as $Z_{2}$ are also common in pathway cross-talk, or when pathways converge upon a common endpoint gene.

**Figure 4.**
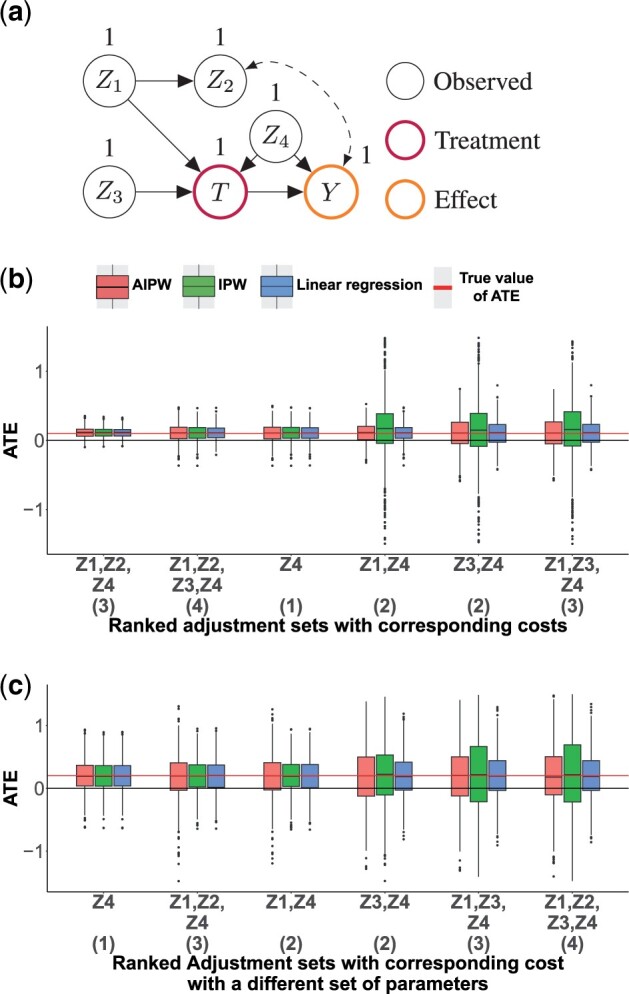
(a) Case study 1. ADMG weighted by cost. (b) The estimates of ATE with AIPW, IPW, and linear regression estimators over *K* datasets. In parentheses is the measurement cost of each adjustment set. True data generating process parametrized by θ. (c) As in (b), with true data generating process parametrized by $\theta^{\prime }$.

Denote X the set of all the variables. Assume that, for a fixed set of parameters θ, the data generation process is:


(2) $$X=\theta Pa(X)+N_{X};N_{X}\overset{\mathrm{iid}}{∼}N(0,1).$$

The causal query of interest, ATE, is the coefficient of *T* obtained by regressing *Y* on *T* and on an adjustment set. We refer to data generated from (2) as **historical data**.

In graphical structures that contain non-ancestors of the treatment and effect (Rotnitzky and Smucler 2022) such as $Z_{2}$ in Fig. 4a, different values of θ produce different optimal adjustment sets (Henckel *et al.* 2021). Therefore, **in this Case study no state-of-the-art approaches using a graphical criterion can find the optimal adjustment set.**

To illustrate that, we first considered the true data generating process with parameters θ∼Uniform(−5,5), such that the optimal adjustment set was $\left\{Z_{1},Z_{2},Z_{4}\right\}$, and the rankings of rest of valid adjustment sets was as shown in x-axis of Fig. 4b. Second, we considered a different true data generating process with parameters $\theta^{\prime }∼\mathrm{Uniform}(-5,5)$, such that the optimal adjustment set was $Z_{4}$, and the rankings of rest of valid adjustment sets was as shown in *x*-axis of Fig. 4c. The Case study illustrates that Algorithm 1 accurately determined the optimal adjustment set for each θ.

**Input:** The proposed approach took as input the weighted ADMG in Fig. 4a (with the measurement cost of all the variables set to 1), the causal query (ATE), and set the number of synthetic datasets K=1000. The proposed approach did not take as input the true data generation process, but the historical dataset with N=1000.

**Step 1: simulating future datasets:** We approximated the true data generating process with a Bayesian network, using parametrized generalized linear models (GLMs). At each node the GLMs had a Normal distribution with identity link, except for the treatment node which had a Binomial distribution with logit link. The model was fit on the historical data, using Bayesian Information Criterion (BIC) to guide feature engineering and transformations. Once the model was fit, *N* new samples were generated by forward simulation. We repeated the forward simulation *K* times to generate multiple synthetic datasets.

**Step 2: exploring adjustment sets:** The Case study had six valid adjustment sets $\left\{Z_{4}\right\}$, $\left\{Z_{1},Z_{4}\right\}$, $\left\{Z_{3},Z_{4}\right\}$, $\left\{Z_{1},Z_{3},Z_{4}\right\}$, $\left\{Z_{1},Z_{2},Z_{4}\right\}$, and $\left\{Z_{1},Z_{2},Z_{4},Z_{4}\right\}$. While the exploration identified $Z_{4}$ as a minimal adjustment set (i.e. $Z_{4}$ alone blocked the backdoor paths between *T* and *Y*), the optimal adjustment set remained unknown.

**Step 3: estimating causal query:** For each simulated dataset and each valid adjustment set, we estimated the ATE with linear regression estimator, IPW, and AIPW estimators.

**Output and conclusions:** Figure 4b and c summarize the query estimates for each valid adjustment set over *K* datasets, for the two sets of θ. The figure makes three points. First, the choice of the adjustment set mattered, as different adjustment sets produced different distributions of the estimates. Second, the optimal adjustment set depended on the data generation process. Finally, while the optimal adjustment set produced estimates of ATE away from zero, the distribution of the estimates with less optimal but valid adjustment sets included zero. In other words, less optimal adjustment sets increased the uncertainty of the estimation, and could not conclusively detect the treatment effect.

We repeated the experiment with N=15,20,50,100,500. Starting from N=20, the ranking of the adjustment sets remained consistent across values of *N*, the data generation processes, and the estimators.

**Comparison with state-of-the-art:** The graph structure in this Case study has non-ancestors of the treatment and effect, and supports multiple data generation processes. To the best of our knowledge, none of the state-of-the-art approaches utilizing graphical criteria can find the optimal adjustment set in this situation. The state-of-the-art graphical criteria could determine the minimal adjustment set ($Z_{4}$), however it was not optimal for θ in Fig. 4b.

### 4.2 Case study 2: the IGF signaling pathway

**Overall objective:** This Case study investigates the robustness of the proposed approach (Algorithm 1) to two different synthetic data generation strategies.

**Ground truth:** Figure 5a is the weighted ADMG of the insulin growth factor (IGF) signaling, regulating growth and energy metabolism of a cell. Variables are kinase proteins.

**Figure 5.**
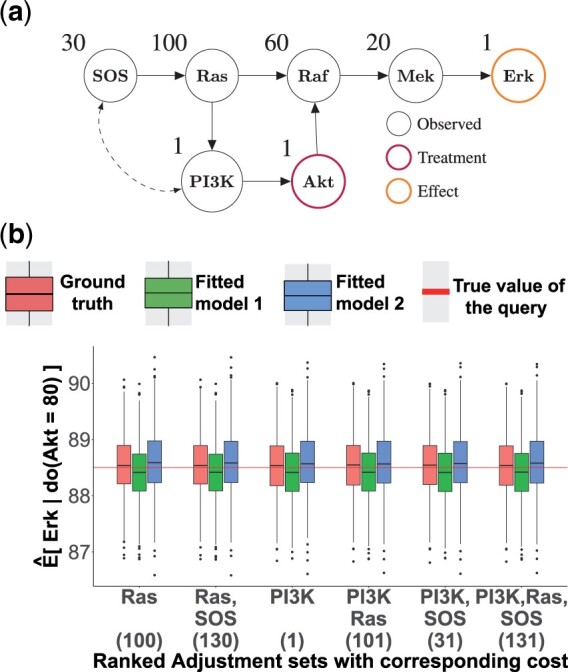
(a) Case study 2. ADMG weighted by cost. (b) Ranked adjustment sets. In parentheses is the measurement cost of each adjustment set. The variances of the adjustment sets were indistinguishable for all practical purposes. Hence, the optimal adjustment set minimized the experimental cost.

IGF dynamics is well characterized in form of stochastic differential equations (SDE). Data from the true data generation process were collected by setting the initial amount of each protein to 100, and generating subsequent observations via the Gillespie algorithm (Gillespie 1977) with the *smfsb* (Wilkinson *et al.* 2018) R package. We refer to this dataset as **historical dataset**. We further simulated interventional data while fixing Akt=80.

Since for a fixed θ the true data generation process is known, we also know the optimal adjustment set, and the rankings of all the valid adjustment sets (*x*-axis in Fig. 5b).

**Input:** The proposed approach took as input the weighted ADMG in Fig. 5a. The causal query of interest was E[Erk|do(Akt=80)] The proposed approach did not take as input the true data generation process, but the historical dataset above. We set number of synthetic datasets K=1000, and number of synthetic observations N=1000.

**Step 1: simulating future datasets:** We approximated the true and unknown data generation process with two models. The first model was as in Case study 1, where we fit a Bayesian network to the historical dataset.

The second model also used a Bayesian networks with exogenous variables following $N(\mu_{r},\sigma_{r})$. Each non-exogenous variable *X* was modeled with a Hill equation $X∼N(\frac{100}{1+\text{exp}(\theta^{\prime }Pa(X)+\theta_{0})},\sigma_{X})$, with Pa(X) a q×1 vector of parents of *X*, and parameters $\theta_{0}$ (a scalar) and $\theta^{\prime }$ (a 1×q vector). The parameters of the Hill equation were estimated from the historical data with RStan (Stan Development Team 2020), and new samples were generated by forward simulation.

We refer to K=1000 synthetic datasets with N=1000 observations generated from these models as **synthetic data 1** and **synthetic data 2**, respectively.

**Step 2: exploring valid adjustment sets:** This example had six valid adjustment sets, {PI3K}, {Ras}, {PI3K,Ras}, {PI3K,SOS}, {Ras,SOS}, and {PI3K,Ras,SOS}.

**Step 3: estimating causal query:** Since the treatment *Akt* was continuous, IPW and AIPW approaches were not applicable. Hence, we used the linear regression estimator. We refer to the fitted model that used synthetic datasets 1 or 2 as **fitted model 1** and **fitted model 2**, respectively.

**Output and conclusions:** Figure 5b summarizes the query estimates for the valid adjustment sets over *K* datasets, estimated from the ground truth data generating process and interventional data, as well as with the proposed approach models 1 and 2.

We can make several conclusions. First, although the true data generating process was complex and unknown, its approximations by models 1 and 2 produced estimates similar to those generated from the ground truth data generating process. Therefore, approximating the complex process with simpler models was sufficient for the purpose of ranking the adjustment sets. Second, despite the non-linear nature of the true regulatory relationships in the network, the linear regression estimator produced estimates similar to those generated from the ground truth data generating process. Therefore, estimating the ATE with a simpler estimator was sufficient for ranking the adjustment sets. Finally, the variance of the optimal adjustment set and the rest of the valid adjustment sets were indistinguishable for all practical purposes. Hence, the optimal adjustment set minimized the experimental cost. Specifically, since measuring the optimal adjustment set (*Ras*) was costly, a less optimal adjustment set (*PI*3*K*) provided a similar precision at a smaller cost.

We repeated the experiment with N=15,20,50,100. Starting from N=20, the ranking of the adjustment sets remained consistent across values of *N*, and across the models used to approximate the true data generating process.

**Comparison with state-of-the-art:** Since the Case study did not include non-ancestors of the treatment and effect, the optimal adjustment set with respect to asymptotic variance could be detected based on the graphical criterion in (Rotnitzky and Smucler 2022). The output of this criterion was the adjustment set {RAS}. However, the criterion could not rank the remaining valid adjustment sets.

### 4.3 Case study 3: reduced *Escherichia coli* network

**Overall objective:** This Case study showcases the application of Algorithm 1 in a small-scale transcriptional *E.coli* regulatory network (Fig. 6) with an experimental dataset.

**Figure 6.**
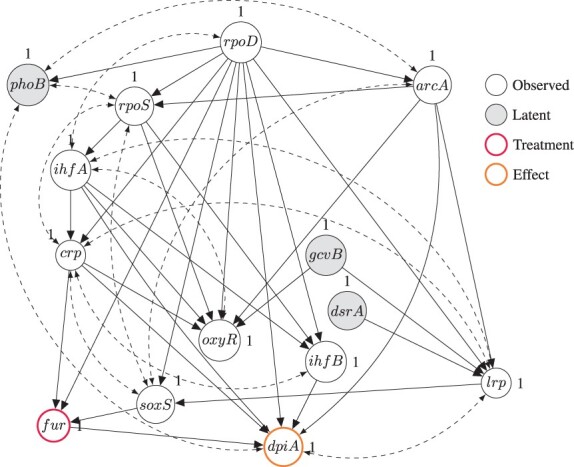
Case study 3. ADMG of the reduced *E.coli* K-12 transcriptional network. The cost of each variable is set to 1.

**Experimental data:** The experimental data were 260 RNA-seq normalized expression profiles of *E.coli* K-12 MG1655 and BW25113 across 154 unique experimental conditions, extracted from the PRECISE database (Sastry *et al.* 2019). We refer to this dataset as **historical observational dataset**.

In addition, the database included three replicates of a gene perturbation experiment, with the value of *fur* set to zero. We refer to this dataset as the **interventional data**. To evaluate the ability to estimate E[dpiA|do(fur=0)], we compared the value reported by the proposed approach to the mean of the effect (*dpiA*) over the three replicates from the interventional data, which was 4.9.

**Input:** The *E.coli* regulatory network was extracted from the EcoCyc database (Keseler *et al.* 2021). To evaluate the appropriateness of the network for this particular experiment, we derived testable conditional independencies from the network, and tested them against the historical observational dataset with the dagitty package (Textor *et al.* 2016). Many of the conditional independence failed. To address this issue, we added a bi-directed edge between all the pairs of variables where the conditional independencies had failed. After that all the tests for conditional independencies passed. Finally, to create input to the proposed approach, the network augmented with bi-directional edges was transformed into the weighted ADMGs in Fig. 6, with all weights set to 1. The query of interest was $Q_{\mathrm{fur}}=E(\mathrm{dpiA}|do(\mathrm{fur}=0))$, K=1000, and N=260.

**Step 1: simulating future datasets:** We approximated the true data generating process with Tabular GAN (Xu *et al.* 2019). We trained the Tabular GAN on historical observational experimental data, while prepossessing and training the data with default hyperparameters as described in Ashrapov (2020). Once the model was fit, *N* new samples were generated by forward simulation. We repeated the simulation *K* times to generate multiple synthetic datasets.

**Step 2: exploring adjustment sets:** The dagitty package reported 64 valid adjustment sets (see section A in Appendix).

**Step 3: estimating causal query:** Since the treatment *fur* was continuous, IPW and AIPW approaches were not applicable. Hence, we used the linear regression estimator.

**Output and conclusions:** Figure A1 in Appendix shows the rankings and the corresponding variance of all the valid adjustment sets. The estimate $\hat{E}(\mathrm{dpiA}|do(\mathrm{fur}=0))$ across all the valid adjustment sets with linear regression estimator was 4.81, which was close to the real value of 4.9 from the experimental interventional data.

We can make several conclusions: First, despite the non-linear regulatory relationships in the experimental data, the results of the linear regression estimator were close to the experimental interventional value. Second, similar to Case study 2, the variance of the optimal adjustment set and of the rest of the valid adjustment sets were very similar. This was useful information, as in this case the choice of the adjustment set could be made based on the cost alone.

**Comparison with state-of-the-art:** Since the network included non-ancestors of the treatment and effect (*phoB*), to the best of our knowledge none of the state-of-the-art approaches based on graphical criteria can find the optimal adjustment. The existing approaches could identify the minimum adjustment set, but were unable to compare its variance to other valid adjustment sets. In contrast, the proposed approach established the fact that all the valid adjustment sets had similar variances.

### 4.4 Case study 4: extended *Escherichia coli* network

**Overall objective:** This Case study showcases the scalability of Algorithm 1 to a larger-scale transcriptional *E.coli* regulatory network with an experimental dataset.

**Experimental data and analysis:** This Case study uses the same setup and analysis steps as in Case study 3, but with a larger-scale transcriptional *E.coli* regulatory network with 52 nodes and 146 edges obtained from EcoCyc database (Keseler *et al.* 2021). The input to Algorithm 1 is an ADMG. Hence, first, we transformed the regulatory network to an ADMG according to the simplification rules in Section 2.1. In addition, step 2 of Algorithm 1 will remove all the descendants of the output (*dpiA*) from the ADMG. As the result, the size of the final ADMG was reduced to 16 nodes and 61 edges and contained 2,336 valid adjustment sets. In Section B of the Appendix, Fig. B1 displays the original network.

**Output and conclusions:** Figure B2 in Appendix shows the ranking and the corresponding variance of selected adjustment sets. The estimate $\hat{E}(\mathrm{dpiA}|do(\mathrm{fur}=0))$ across all the valid adjustment sets with linear regression estimator was 4.83, a value close to both the estimate in Case study 3 and to the experimental interventional value 4.9.

We can make several conclusions. First, the overall conclusions regarding the proposed approach were as in Case study 3. Second, despite the exponential nature of Algorithm 1, the proposed approach ranked the adjustment sets within an hour of time. Finally, the accuracy of the causal query estimator for the larger-scale network in Case study 4 was similar to that of for the smaller-scale network in Case study 3, indicating that a smaller network may be sufficient in some cases.

### 4.5 Summary of the conclusions of the case studies

**In presence of latent variables, the optimal adjustment set may depend on the data generation process:** In Case studies 1, 3, and 4, the optimal adjustment set could not be determined based on a graphical criterion alone. Case study 1 showed that different data generating processes produce different rankings of valid adjustment sets, even when supported by a same graphical structure. Therefore, it is important to account for the data generating process when selecting adjustment sets.

**The proposed approach produced accurate rankings of valid adjustment sets:** In Case studies 1 and 2, reasonable approximations to the true data generating process were sufficient to produce accurate ranking of the adjustment sets across different estimators. Therefore, approximating the true data generating process with an appropriate model, fitting this model to a historical observational dataset, and generating synthetic data is a useful approach for exploring and selecting adjustment sets.

**Multiple adjustment sets may have a similar variance of the causal query estimator:** The variances of multiple valid adjustment sets in Case studies 1, 3, and 4 were very similar. These situations are useful, as they allow experimentalists to select an adjustment set based on cost.

**Different models of the true data generating process were appropriate for different networks:** Bayesian networks were appropriate models of the data generating processes in Case studies 1 and 2. In contrast, Tabular GAN were more appropriate for Case studies 3 and 4. The choice was not arbitrary. The data generating process in Case studies 1 and 2 was not too different from a linear model, and in these situations GAN overfit. In Case studies 3 and 4, the true data generation process was more complex, and Tabular GAN were more successful. Model accuracy can be verified with sensitivity analysis, by comparing the marginal and pairwise relationships of the variables from synthetic data with the historical experimental data.

**The proposed approach scaled well to larger biomolecular network:** Despite the exponential nature of the proposed approach, a larger network in Case study 4 produced query estimate of a similar accuracy as a smaller network. The results were obtained within an hour on a single Google cloud platform. The scope of the proposed approach can be expanded to even larger biological scenarios with additional computational resources.

## 5 Discussion

We proposed an approach for selecting optimal adjustment sets for causal query estimation. Its potential limitation is the requirement of a known biomolecular network. We overcame this limitation in part by working with ADMGs, which allow us to misspecify the latent variables as long as the observed variables are represented accurately. The correctness of the structure over observed variables can be checked with the falsification modules, such as in $Y_{0}$ (Zucker and Hoyt 2021) or dagitty. Another potential limitation is the accuracy of the model that approximates the true data generating process, which can be addressed by sensitivity analysis. Finally, although the complexity of the proposed approach is exponential, it scales successfully to larger biomolecular networks. This is due to the fact that many of the nodes in large networks (such as the descendants of the outcome) are irrelevant for estimating the causal query, and can be ignored. In addition, Evan’s simplification rules help further reduce the size of the network. Future directions of this research include a closer integration with simulated interventional data. This will allow us to expand the scope of causal queries, check their idenifiability, e.g. with the G-ID algorithm (Lee *et al.* 2020), and increase their practical use.

## Figures and Tables

**Figure A1.**
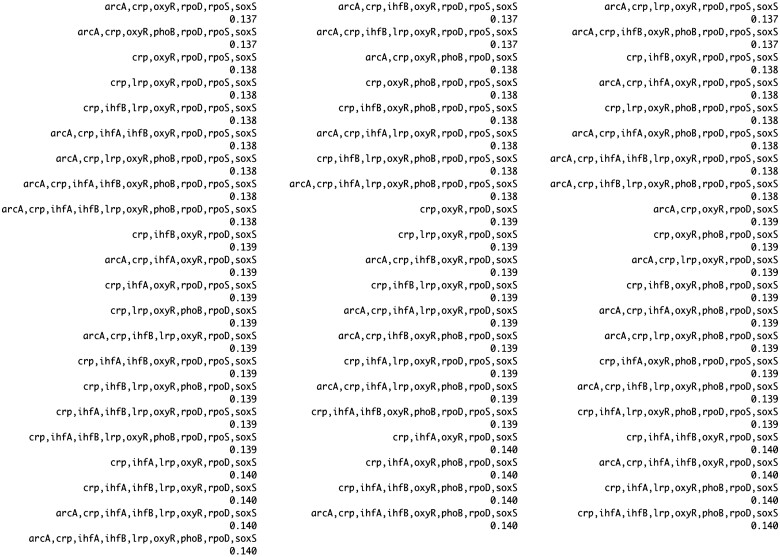
Case study 3. Ranked adjustment sets of the small *E.coli* case study with their corresponding variance.

**Figure B1.**
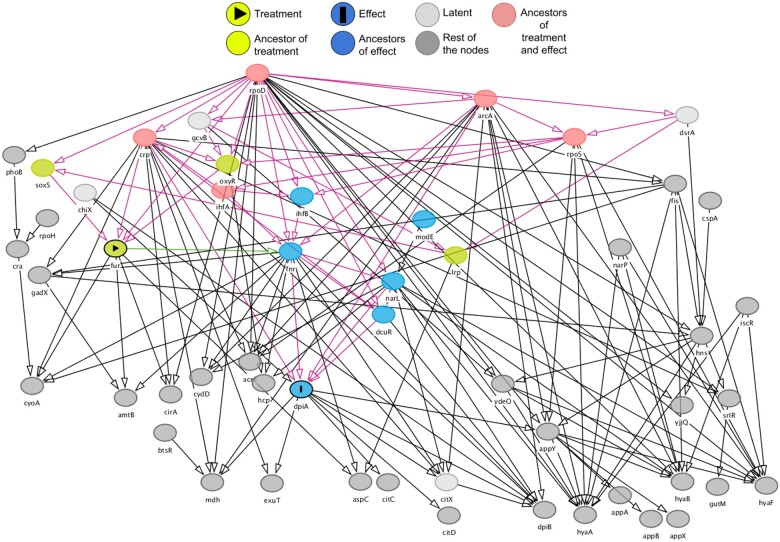
Case study 4. Weighted ADMG for the extended *E.coli* K-12 transcriptional network. The cost of each variable is set to 1.

**Figure B2.**
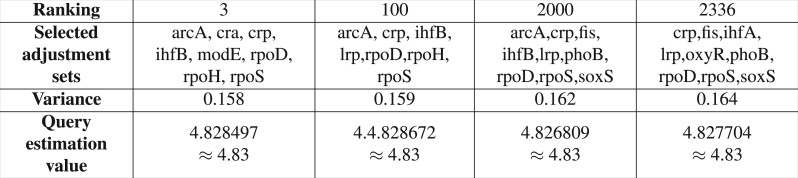
Case study 4. Selected rankings of the valid adjustment sets with their corresponding variance. The cost of measuring each variable is 1, hence the cost of an adjustment set is the total number of its variables. The query estimation value is $\hat{E}(\mathrm{dpiA}|do(\mathrm{fur}=0))$.

## Appendix

### A: Case study 3

Figure 7 shows all the valid adjustment sets with their corresponding variance for the small *E.coli* case study.

### B: Case study 4: extended *Escherichia coli* K-12 transcriptional network

Figure B1 shows the weighted ADMG for the extended *E.coli* K-12 transcriptional network. Figure B2 shows selected valid adjustment sets from the large *E.coli* example with their corresponding variance.
