# Correction to: Optimal adjustment sets for causal query estimation in partially observed biomolecular networks

**DOI:** 10.1093/bioinformatics/btad559

**Published:** 2023-09-13

This is a correction to: Sara Mohammad-Taheri and others, Optimal adjustment sets for causal query estimation in partially observed biomolecular networks, *Bioinformatics*, Volume 39, Issue Supplement_1, June 2023, Pages i494–i503, https://doi.org/10.1093/bioinformatics/btad270

In the originally published version of this manuscript, the sixth author’s name was incorrectly spelled as Charles Taply Hoyt. It should be Charles Tapley Hoyt.

This error has been corrected online.

